# Prevalence and Factors Influencing Use of Internet and Electronic Health Resources by Middle-Aged and Older Adults in a US Health Plan Population: Cross-Sectional Survey Study

**DOI:** 10.2196/11451

**Published:** 2019-03-26

**Authors:** Elizabeth Crouch, Nancy P Gordon

**Affiliations:** 1 Department of Health Services Policy and Management University of South Carolina Columbia, SC United States; 2 Division of Research Kaiser Permanente Northern California Oakland, CA United States

**Keywords:** digital divide, patient portal, information-seeking behavior, health education, patient preference, patient surveys

## Abstract

**Background:**

Health care organizations are increasingly using electronic health (eHealth) platforms to provide and exchange health information and advice (HIA). There is limited information about how factors beyond internet access affect use of eHealth resources by middle-aged and older adults.

**Objective:**

We aimed to estimate prevalence of use of the internet, health plan patient portal, and Web-based HIA among middle-aged and older adults; investigate whether similar sociodemographic-related disparities in eHealth resource use are found among middle-aged and older adults; and examine how sociodemographic and internet access factors drive disparities in eHealth resource use among adults who use the internet.

**Methods:**

We analyzed cross-sectional survey data for 10,920 Northern California health plan members aged 45 to 85 years who responded to a mailed and Web-based health survey (2014-2015). We used bivariate and multivariable analyses with weighted data to estimate prevalence of and identify factors associated with internet use and self-reported past year use of the health plan’s patient portal and Web-based HIA resources by middle-aged adults (aged 45 to 65 years; n=5520), younger seniors (aged 65 to 75 years; n=3014), and older seniors (aged 76 to 85 years; n=2389).

**Results:**

Although approximately 96% of middle-aged adults, 92% of younger seniors, and 76% of older seniors use the internet to obtain information, about 4%, 9%, and 16%, respectively, require someone’s help to do so. The percentages who used the patient portal and Web-based HIA resources were similar for middle-aged adults and younger seniors but lower among older seniors (59.6%, 61.4%, and 45.0% and 47.9%, 48.4%, and 37.5%, respectively). Disparities in use of the internet, patient portal, and Web-based HIA across levels of education and between low and higher income were observed in all age groups, with wider disparities between low and high levels of education and income among seniors. Multivariable analyses showed that for all 3 age groups, educational attainment, ability to use the internet without help, and having 1 or more chronic condition were significant predictors of patient portal and Web-based HIA use after controlling for gender, race/ethnicity, and internet use.

**Conclusions:**

Internet use, and especially use without help, significantly declines with age, even within a middle-aged group. Educational attainment is significantly associated with internet use, ability to use the internet without help, and use of patient portal and Web-based HIA resources by middle-aged and older adults. Even among middle-aged and older adult internet users, higher educational attainment and ability to use the internet without help are positively associated with patient portal and Web-based HIA use. Organizations serving middle-aged and older adults should take into account target population characteristics when developing and evaluating uptake of eHealth resources and should consider offering instruction and support services to boost patient engagement.

## Introduction

The internet is often used as a source of health information by individuals interested in learning about new diagnoses, options, medications, or healthy behaviors [1-3]. Web-based patient portals provide individuals continuous access to test results and other information in their electronic health records and a convenient way to communicate with their health care providers, order medications, and arrange for appointments. Digital information technologies (DITs) can also connect patients and providers without a physical office visit, allowing providers to tailor care to patient-specific needs and preferences [4] and to conduct remote monitoring of health conditions [5,6]. Online support groups, chat rooms, and forums provide the opportunity for peer-to-peer sharing of health information and advice (HIA), as well as potentially serving as a source of support to adults who are socially isolated [7].

Because middle-aged and older adults are more likely to be managing chronic conditions than younger adults [8] or to be caregivers for family members with health problems, individuals in these age groups potentially have the most to gain from using the profusion of health resources available on the internet. It has been well-documented that in the United States, seniors (aged ≥ 65 years) are less likely than younger and middle-aged adults to use the internet, email, and patient portals and to trust the internet as a source of health information [9,10] and that within the senior age group, there are disparities by older age (>75 years), race/ethnicity, education, and income among older adults in use of DIT and specifically use for health-related purposes [9,11-18]. Less is known about the use of DIT and internet-based health information resources by middle-aged adults, who, like seniors, are not *digital natives* as they did not have the opportunity to learn to use computers, smartphones, the internet, and email during childhood [19-22].

In response to Meaningful Use requirements and recognized advantages of using a Web-based platform for secure communications and health information sharing, health care organizations have been investing substantial resources in developing user-friendly patient portals and health information websites, hoping that health plan members will transition to these Web-based resources for exchanging health information. Increasing adults’ access to the internet and use of the internet and digital technologies for health-related purposes are also US governmental goals for the American public, stated in *Healthy People 2020* [23]. Although this transition is probably easy and welcomed by most younger adults, the same should not be assumed for middle-aged and older adults, many of whom did not have the opportunity to use computers, mobile devices, and the internet at school or on the job. Awareness of characteristics of middle-aged and older adult patients that may contribute to lower likelihood of engaging with patient portals and Web-based health information resources, combined with information about the sociodemographic characteristics of middle-aged and older adults in a target patient population, can help health care organizations plan and monitor this transition to electronic health (eHealth) platforms and identify segments of the patient population that may need a higher level of outreach and support to make this transition.

This study had several aims. First, we wanted to estimate and compare prevalence of use of the internet, patient portal, and Web-based health information resources among middle-aged, younger senior, and older senior adult members of a large Northern California, United States, health plan that had a well-established patient portal and health information website. Second, we wanted to examine the extent to which prevalence of the use of Web-based health resources was due to not being an internet user. Third, we wanted to learn whether sociodemographic factors known to be associated with disparities in use of the internet and Web-based health resources among older adults operated similarly among middle-aged adults. Fourth, we wanted to learn whether these sociodemographic factors remained significant predictors of use of Web-based health resources among those who were using the internet alone or with someone’s help. Finally, we wanted to learn whether the ability to use the internet without assistance from another person, having easy access to a computer for using the internet, and having a chronic health condition influenced likelihood of using Web-based health resources beyond sociodemographic factors.

## Methods

### Setting

Kaiser Permanente in Northern California (KPNC) is a vertically integrated health care delivery system that serves over 2.5 million adult members who mostly reside in the San Francisco Bay Area, Silicon Valley, Sacramento area, or Central Valley, California, United States. The sociodemographically diverse KPNC adult membership is very similar to the insured population of Northern California with regard to demographic and health characteristics [24]. KPNC has a comprehensive website that provides information about health plan benefits and resources and health information (eg, about health conditions, medications, and healthy behaviors/lifestyle) that is accessible to both members and the general public and a secure patient portal that is only available to health plan members who register for and activate a patient portal account. For several years, the health plan has encouraged members to obtain HIA and communicate with health care providers using its website and patient portal.

### Survey Sample

This study used data obtained from 5520 middle-aged adults (aged 45 to 65 years), 3014 younger seniors (aged 66 to 75 years), and 2389 older seniors (aged 76 to 85 years) who responded to the 2014/2015 cycle of the KPNC Member Health Survey (MHS). The MHS is a self-administered (mailed print and Web-based) survey that has been sent to independent stratified random samples of adults every 3 years beginning in 1993. The survey, which is only conducted in English, captures information about sociodemographic and health-related characteristics as well as access to different electronic modes of communication, sources used to obtain health information in the past year, and interest in using different modalities to obtain HIA. More information about the survey is available in an earlier publication [9] and on the survey website [25]. In the 2014/2015 cycle, the overall response rate was 49.3% for members aged between 45 and 85 years (40.9% for those aged between 45 and 65 years and 64.5% for those aged between 66 and 85 years).

### Study Variables

#### Sociodemographic Characteristics

Age group (45 to 65, 66 to 75, and 76 to 85 years for age group comparisons; 45 to 55, 56 to 65, 66 to 70, 71 to 75, 76 to 80, and 81 to 85 years for regression models), gender (female and male), race/ethnicity (white, black, Latino, Filipino, East Asian, other Asian, Pacific Islander, and other), educational attainment (<high school graduate, high school graduate/GED/technical school, some college, and college graduate), and household income (US $35,000 to $50,000, $50,001 to $65,000, $65,001 to $80,000, $80,001 to $100,000, and >$100,000).

#### Health Characteristics

Self-rated health (excellent/very good, good, and fair/poor) and 1 or more chronic health condition in the past 12 months (diabetes, high blood pressure, heart condition, cancer, chronic obstructive pulmonary disease (COPD), chronic pain, severe musculoskeletal pain, severe headaches or migraines, depression, anxiety, and insomnia).

#### Use of Digital Technology

Uses the internet to get information from websites, uses the internet without help from another person, and has access to a computer or tablet if they want to use one.

#### Use of Web-Based Health Resources in Past 12 Months

Patient portal users were those who indicated having used the health plan’s patient portal to email clinicians, view lab results, or refill prescriptions. Users of Web-based health information (HIA) resources were those who reported having obtained HIA from any website, using Web-based patient education programs (eg, preparing for a procedure, health calculator, or health lifestyle programs for nutrition, weight, stress, or exercise) or podcasts found on the health plan’s website, or participating in an online chat room or community related to a health condition. Adults who had used Web-based HIA resources and/or the patient portal were considered to be eHealth or Web-based health resource users.

### Data Analysis

All analyses were performed using SAS version 9.4 procedures for data from complex survey designs (SAS Institute, Cary, NC 2014) and data weighted to the age, gender, and geographic composition of the KPNC adult membership in 2014. Proc Surveyfreq was used to produce weighted percentages for our 4 outcomes of interest (use of the internet to obtain information, use of the internet without someone’s help, use of the health plan’s patient portal in the past year, and use of Web-based HIA resources in the past year) overall and by sociodemographic and health characteristics. We reported 95% margins of error around the estimated percentages that correspond to a 95% CI when added to or subtracted from the percentage estimate.

We examined the bivariate relationships of sociodemographic and health characteristics with these outcomes using bivariate Proc Surveylogistic models to test for statistically significant differences between subgroups as compared with a referent subgroup for each outcome. We then used multivariable logistic regression models to examine the independent association of these characteristics with each of the 4 outcomes after adjusting for the other characteristics. All logistic regression models included indicator variables for age group (reference group: aged 45 to 55 years for middle-aged, 66 to 70 years for young seniors, and 76 to 80 years for older seniors), gender (reference group: male), race/ethnicity (reference group: white non-Hispanic), education (reference group: college graduate), and income (reference group: ≤ $35,000). Models that predicted use of the health plan’s patient portal and Web-based HIA resources during the previous year additionally used indicator variables for internet use status (reference group: uses internet by self, compared with no internet use to obtain information and use with someone’s help), easy access to a computer (reference group: lacks easy computer access), and having 1 or more chronic condition (reference group: none of the chronic conditions) in addition to the sociodemographic factors. Indicator variables for unknown education and income were included in all models so that results would be based on the full age group. In supplemental files, we reported the results of multivariate logistic regression models of patient portal and Web-based HIA resource use that did not include the internet use of computer variables. All differences between subgroups mentioned in the text are statistically significant at *P*<.05 or greater; if differences are not mentioned, they did not reach that threshold. Although we did not adjust for multiple comparisons, we have reported results of all statistical comparisons.

### Ethics

This study was approved by KPNC’s Institutional Review Board.

## Results

### Characteristics of Survey Respondents

Table 1 provides a description of the characteristics of the middle-aged, younger senior, and older senior groups.

**Table 1:** Characteristics of middle-aged and older adult study groups.

Characteristics		45 to 65 years (n=5520), n (%)^a^	66 to 75 years (n=3014), n (%)	76 to 85 years (n=2389), n (%)
**Age (years)**				
	45-55	2563 (54.9)	—^b^	—
	56-65	2957 (45.1)	—	—
	66-70	—	1521 (60.2)	—
	71-75	—	1493 (39.8)	—
	76-80	—	—	1956 (57.7)
	81-85	—	—	433 (42.3)
**Gender**				
	Male	2551 (47.7)	1482 (46.1)	1268 (45.0)
	Female	2969 (52.3)	1532 (53.9)	1121 (55.0)
**Race/ethnicity**				
	White non-Hispanic	3214 (58.4)	2109 (71.6)^c^	1695 (74.1)^c^
	Black	429 (7.0)	195(6.0)	147 (6.0)
	Hispanic	763 (13.6)	242 (7.2)^b^	238 (8.7)^b^
	Filipino	312 (5.5)	160(4.8)	107 (3.7)
	East Asian	509 (9.2)	176 (5.7)^c^	133 (4.9)^c^
	Other Asian	143 (3.5)	59 (2.4)	27 (0.8)
	Other	150 (2.8)	73 (2.3)	42 (1.8)
**Education**				
	<12 years	143 (2.5)	117 (3.1)	188 (8.9)^c,d^
	High school graduate/General Education Development	1025 (18.7)	596 (18.4)	654 (29.8)
	Some college/AA degree	1878 (33.6)	1023 (35.1)	739 (29.9)
	College graduate	2444 (45.2)	1250 (43.4)	781 (31.3)^c,d^
**Household income (US $)**				
	<$25,000	389 (7.1)	355 (11.6)^c^	469 (24.5)^c,d^
	$25,001-$35,000	273 (4.6)	300 (10.5)	292 (14.0)
	$35,001-$50,000	547 (9.9)	413 (14.4)	402 (20.4)
	$50,001-$65,000	527 (9.6)	328 (11.9)	278 (11.9)
	$65,001-$80,000	639 (12.0)	365 (13.7)	233 (10.1)
	$80,001-$100,000	800 (14.9)	360 (13.9)	192 (8.6)
	>$100,000	2084 (41.9)	639 (23.9)^c^	254 (10.4)^c,d^
**Health Status**				
	Excellent/very good	2932 (54.1)	1400 (48.3)^c^	815 (33.4)^c,d^
	Good	1956 (35.6)	1152 (37.5)	1092 (45.7)
	Fair/poor	627 (10.3)	454 (14.2)^c^	474 (20.8)^c,d^
**More than one chronic condition^e^**				
	No	1909 (35.9)	621 (21.6)	369 (15.2)
	Yes	3611 (64.1)	2393 (78.4)^c^	2020 (84.8)^c,d^
**Uses the internet to get information from websites**				
	Does not use	249 (3.9)	382 (10.6)^c^	651 (31.1)^c,d^
	Uses with someone’s help or someone uses it for them	231 (4.0)	280 (8.6)	368 (15.7)
	Uses by self	5038 (92.0)	2350 (80.8)^c^	1365 (53.2)^c,d^
**Has easy access to a computer**				
	No	222 (3.6)	294 (8.2)	501 (24.2)
	Yes	5276 (96.4)	2688 (91.8)^c^	1848 (75.8)^c,d^

^a^n: unweighted count; %: percentage of age group with this characteristic based on weighted survey data.

^b^Not applicable.

^c^Significantly different from the 45 to 65 age group at *P*<.001.

^d^Significantly different from the 66 to 75 age group at *P*<.001.

^e^Chronic condition: diabetes, high blood pressure, heart condition, cancer, COPD/chronic bronchitis, chronic pain, depression, anxiety, or insomnia, based on self-report.

Slightly over half of each age group is female. Compared with the middle-aged group, the 2 senior groups have higher percentages of non-Hispanic whites (approximately 70% vs 58%) and lower percentages of Hispanics (approximately 7% vs 14%) and East Asians (approximately 5% vs 9%), with no difference in percentages of blacks and Filipinos. Although the middle-aged and younger senior groups are similar with regard to educational attainment (>40% college graduates and approximately 20% high school graduates or less), the percentage of older seniors with a college degree is significantly lower (31%) and the percentage with no formal education beyond high school (39%) is significantly higher than the younger groups. The percentage considered lower income for the San Francisco Bay Area (≤$35,000) significantly increases with age (approximately 12%, 22%, and 39%, respectively), whereas the percentages with higher incomes (>$80,000) decreases with age (approximately 57%, 38%, and 19%). The percentage of adults who consider their health to be very good/excellent decreases with age, and the percentages with fair/poor health and 1 or more chronic health condition increase with age.

**Figure 1:**
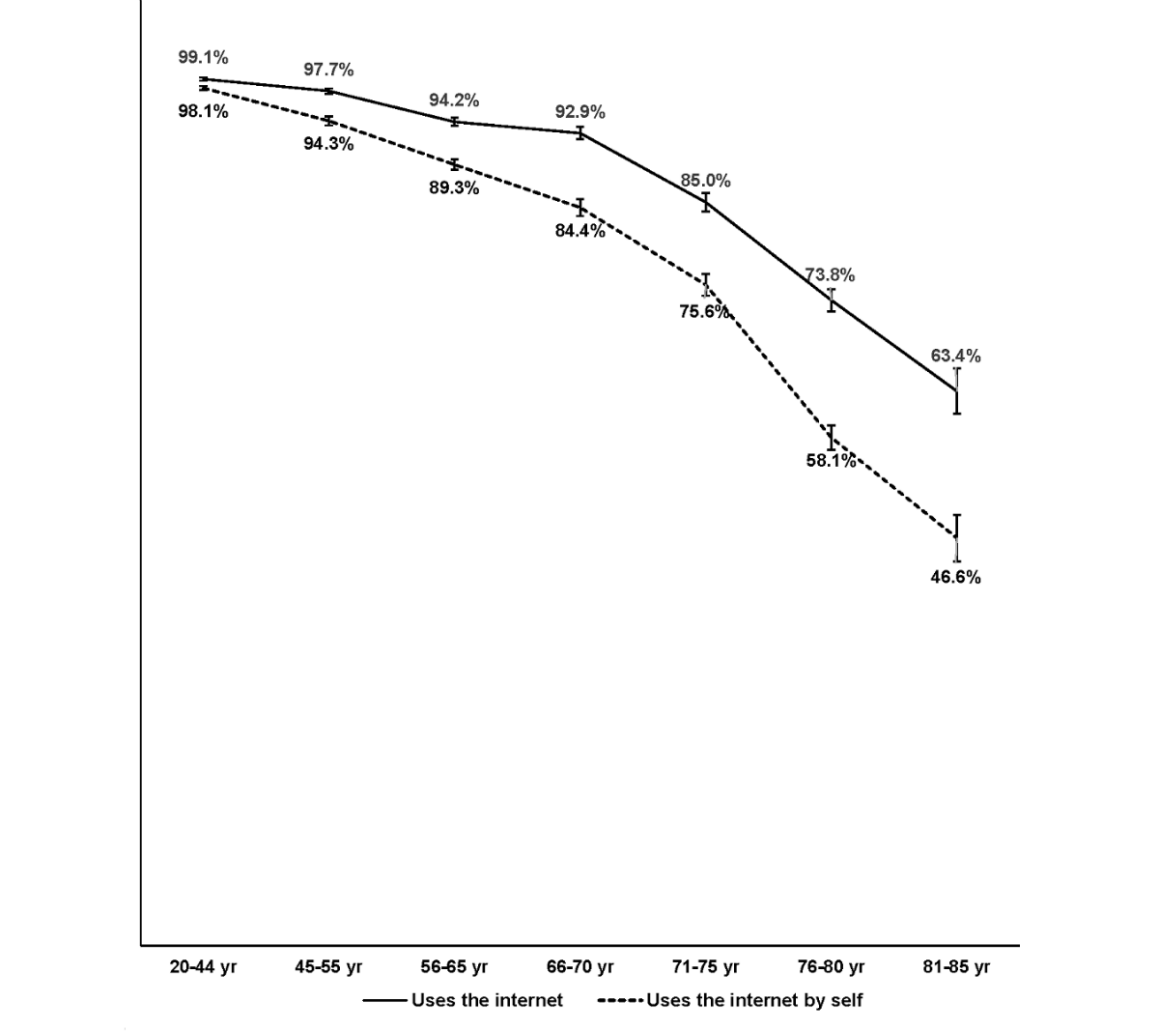
Percentages of adult health plan members aged 20-85 who use the internet to get information from websites, by age group.

Table 1 also shows that easy access to a computer or tablet, use of the internet to obtain information from websites (by oneself or with someone’s help), and use of the internet without someone’s help declines with age group, with younger seniors significantly less likely than middle-aged adults, and older seniors significantly less likely than younger seniors to be using the internet (96%, 89%, and 69%, respectively) and have easy access to a computer (96%, 92%, and 76%, respectively). Figure 1 shows that in this health plan population, middle-aged adults are less likely than younger adults (aged between 20 and 44 years) to be using the internet to obtain information from websites, but also that there is a significant decline within the middle-aged and older adult age groups. Figure 1 also shows that there is an even steeper age-related decline in the ability to use the internet without the help of another person.

### Use of the Internet to Obtain Information

Table 2 shows how internet use to obtain information varies by sociodemographic characteristics within and across the 3 age groups. For each age group, we presented the estimated percentage of internet users in different categories of sociodemographic factors, indicating whether categories of sociodemographic factors significantly differ from the referent group for that factor. We also report the adjusted odds ratios (AOR) for these characteristics after adjusting for the other sociodemographic factors. Within all 3 age groups, blacks, Hispanics, and Filipinos are less likely to be using the internet, whereas the percentages of East Asians who use the internet are similar to whites. In all 3 groups, prevalence of internet use increases with higher educational attainment. Internet use is lower among those with a household income ≤$35,000 compared with >$35,000 but, unlike with education, does not significantly increase at higher levels. In the multivariable models for all 3 age groups, educational attainment, low income, and younger age within that age group remain significant independent predictors of using the internet. In the 2 younger groups, blacks, Hispanics, and Filipinos remain significantly less likely to be internet users, whereas in the older senior group, only Hispanics remain significantly less likely to use the internet. Although the logistic regression models for all 3 age groups show similar AORs for sociodemographic characteristics, comparing percentages across age groups, for both sexes, all race/ethnicities and levels of education, and nearly all income levels, internet use is significantly (*P*<.05 or greater) higher among middle-aged adults than younger seniors and significantly higher among younger seniors than older seniors. In Table 3, the same patterns are observed for use of the internet without another person’s help. However, consistent with Figure 1, the percentages of adults who use the internet by themselves are substantially lower than internet use with or without help, especially among those at the lowest levels of education and income. In Multimedia Appendix 1, we show that significant disparities by race/ethnicity, education, and income persist in the percentages of internet users who use the internet without help.

**Figure 2:**
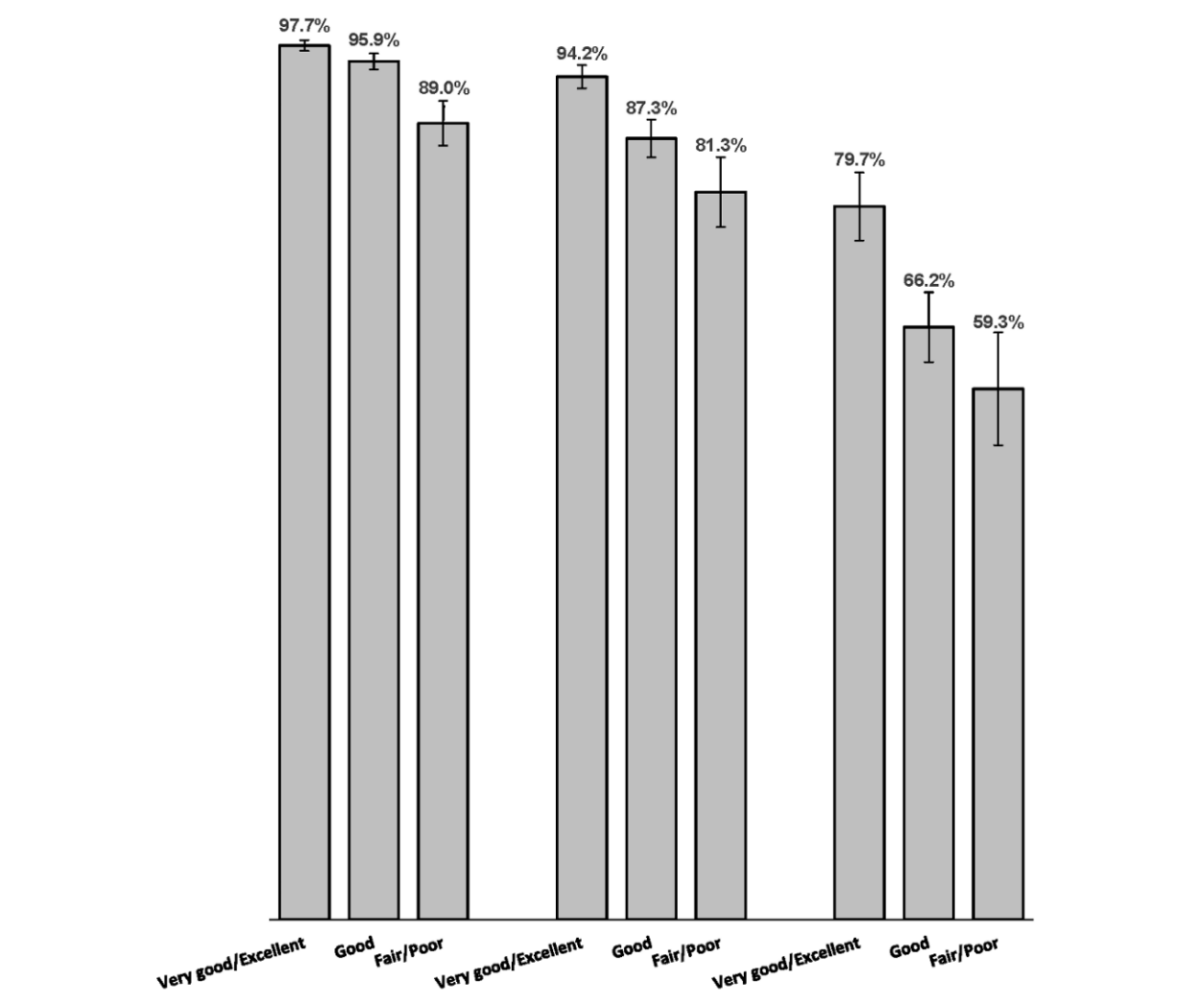
Percentages of middle-aged and older adults who use the internet to get information from websites, by self-rated health.

Figure 2 shows that for all age groups, internet use was higher among adults in excellent/very good health than adults in good health, whereas adults in fair/poor health were less likely to use the internet than those in good health. However, when we controlled for sociodemographic characteristics, health status was not significantly associated with internet use among middle-aged adults. In both senior groups, having excellent/very good health versus good health remained a significant independent predictor of internet use (AOR 1.60, 95% CI 1.17-2.18 for younger seniors and AOR 1.56, 95% CI 1.12-2.16 for older seniors), but fair/poor health was not independently significant. Adults with 1 or more chronic condition were not more likely to use the internet than those with none of the chronic conditions.

### Use of Web-Based Health Resources in Prior Year

The percentages of adults in each age group who used Web-Based health resources during the previous year are shown in Table 4 **.** In contrast to the age group differences observed regarding internet use to get information from websites, middle-aged adults and younger seniors had similar rates of use of Web-Based health resources, with approximately 60% having used the patient portal, 48% having obtained HIA from a Web-based source, and 68% having used at least one of these eHealth resources. The older seniors were significantly less likely than both younger groups to have used the patient portal (52%), Web-based HIA (45%), or either Web-based resource (52%). When we examined eHealth use restricted to internet users, the difference between older seniors and the middle-aged adults goes away, and the younger senior group has significantly higher percentages using these Web-based resources than the middle-aged and older senior groups.

**Table 2:** Sociodemographic characteristics differentiating middle-aged and older adults who report using the internet with or without someone’s help to get information from websites.

Characteristic		45 to 65 years, weighted % (MoE^a^)	66 to 75 years, weighted % (MoE)	76 to 85 years, weighted % (MoE)	45 to 65 years, AOR^b^ (95% CI)	66 to 75 years, AOR (95% CI)	76 to 85 years, AOR (95% CI)
**Age group**							
	Younger age^c^ (ref^d^)	97.7 (0.6)	92.9 (1.4)	73.8 (2.5)	(ref)	(ref)	(ref)
	Older age^c^	94.2 (0.9)^e^	85.0 (2.0)^e^	63.4 (5.1)^e^	0.37 (0.26-0.52)^e^	0.51 (0.39-0.68)^e^	0.66 (0.50-0.88)^f^
**Gender**							
	Male (ref)	95.7 (0.8)	89.9 (1.7)	75.6 (3.1)	(ref)	(ref)	(ref)
	Female	96.6 (0.7)	89.7 (1.6	64.2 (3.9)^e^	1.69 (1.25-2.29)^e^	1.29 (0.97-1.73)	0.83 (0.64-1.07)
**Race/Ethnicity**							
	White non-Hispanic (ref)	97.4 (0.6)	92.4 (1.2)	72.9 (3.0)	(ref)	(ref)	(ref)
	Black	91.9 (2.6)^e^	78.8 (6.3)^e^	63.2 (11.2)	0.45 (0.29-0.70)^e^	0.42 (0.27-0.67)^e^	0.82 (0.45-1.49)
	Hispanic	93.2 (1.9)^e^	81.2 (5.2)^e^	46.4 (8.0)^e^	0.58 (0.38-0.88)^f^	0.53 (0.35-0.81)^f^	0.52 (0.35-0.76)^e^
	Filipino	93.8 (2.6)^e^	76.0 (7.4)^e^	62.0 (11.8)	0.35 (0.12-0.99)^f^	0.11 (0.03-0.38)^e^	0.99 (0.30-3.28)
	East Asian	98.0 (1.2)	89.3 (4.8)	68.7 (10.2)	0.97 (0.31-3.05)	0.28 (0.08-0.95)^g^	0.98 (0.32-2.99)
**Education**							
	<High school graduate	80.7 (6.3)^e^	57.4 (10.1)^e^	37.5 (8.8)^e^	0.09 (0.04-0.17)^e^	0.09 (0.05-0.17)^e^	0.13 (0.08-0.22)^e^
	High school graduate	90.3 (1.9)^e^	77.4 (3.7)^e^	52.7 (5.3)^e^	0.15 (0.09-0.28)^e^	0.17 (0.11-0.26)^e^	0.18 (0.12-0.27)^e^
	Some college/AA degree	96.5 (0.9)^e^	90.8 (1.9)^e^	75.4 (4.4)^e^	0.38 (0.21-0.68)^e^	0.39 (0.25-0.59)^e^	0.45 (0.29-0.67)^e^
	College graduate (ref)	99.2 (0.4)	96.7 (1.0)	89.1 (3.0)	(ref)	(ref)	(ref)
**Household income (US $)**							
	≤$35,000 (ref)	84.0 (2.9)	76.1 (3.6)	52.8 (4.8)	(ref)	(ref)	(ref)
	$35,001-$50,000	91.2 (2.5)^e^	88.0 (3.4)^e^	73.2 (6.0)^e^	1.90 (1.27-2.86)^f^	2.04 (1.36-3.04)^e^	1.97 (1.36-2.85)^e^
	$50,001-$65,000	97.0 (1.6)^e^	92.6 (2.9)^e^	82.4 (5.7)^e^	5.83 (3.22-10.54)^e^	3.09 (1.86-5.12)^e^	2.83 (1.73-4.64)^e^
	$65,001-$80,000	97.6 (1.3)^e^	94.8 (2.4)^e^	80.5 (6.9)^e^	5.73 (3.18-10.32)^e^	4.20 (2.37-7.45)^e^	2.31 (1.37-3.92)^f^
	$80,001-$100,000	98.7 (0.7)^e^	95.5 (2.1)^e^	85.6 (6.3)^e^	9.95 (5.34-18.56)^e^	3.57 (2.02-6.30)^e^	2.32 (1.30-4.12)^e^
	>$100,000	99.4 (0.3)^e^	96.7 (1.6)^e^	90.0 (4.9)^e^	13.79 (7.44-25.56)^e^	3.81 (2.09-6.94)^e^	3.34 (1.79-6.25)^e^
Model c-statistic^h^		—^i^	—	—	.87	.82	.75

^a^MoE: 95% margin of error around estimated percentage (95% confidence intervals can be created from percentage ± MoE).

^b^AOR: adjusted odds ratio from logistic regression model that includes age group, gender, race/ethnicity (including *Other Asian* and *Other* race/ethnicity categories), education, and household income.

^c^Younger age: 45 to 55 years, 66 to 70 years, 76 to 80 years; Older age: 56 to 65 years, 71 to 75 years, 81 to 85 years.

^d^Ref: reference group for comparison of variable categories.

^e^Significantly differs from reference group at *P*<.001.

^f^Significantly differs from reference group at *P*<.01.

^g^Significantly differs from reference group at *P*<.05.

^h^The model c-statistic assesses how well the full logistic regression model predicts which individuals use the internet to obtain information with or without help from other people.

^i^Not applicable.

**Table 3:** Sociodemographic characteristics differentiating middle-aged and older adults who report using the internet by themselves to get information from websites.

Characteristic		45 to 65 years, weighted % (MoE^a^)	66 to 75 year, weighted % (MoE)	76 to 85 years, weighted % (MoE)	45 to 65 years, AOR^b^ (95% CI)	66 to 75 years, AOR (95% CI)	76 to 85 years, AOR (95% CI)
**Age group**							
	Younger age^c^	94.3 (1.0)	84.4 (1.9)	58.1 (2.7)	(ref^d^)	(ref)	(ref)
	Older age^c^	89.3 (1.2)^e^	75.6 (2.4)^e^	46.6 (5.2)^e^	0.46 (0.36-0.59)^e^	0.68 (0.54-0.85)^e^	0.64 (0.49-0.85)^e^
**Gender**							
	Male (ref)	90.8 (1.2)	81.4 (2.1)	58.6 (3.5)	(ref)	(ref)	(ref)
	Female	93.2 (1.0)^f^	80.4 (2.1)	48.9 (4.0)^e^	1.86 (1.46-2.36)^e^	1.19 (0.94-1.49)	0.96 (0.74-1.23)
**Race/Ethnicity**							
	White non-Hispanic (ref)	94.5 (0.8)	85.4 (1.6)	58.5 (±3.2)	(ref)	(ref)	(ref)
	Black	86.1 (±3.5)^e^	70.2 (7.2)^e^	39.0 (±10.6)^e^	0.49 (0.34-0.71)^e^	0.57 (0.38-0.85)^f^	0.54 (0.32-0.89)^g^
	Hispanic	85.9 (2.7)^e^	69.3 (6.3)^e^	29.1 (6.7)^e^	0.58 (0.42-0.79)^e^	0.59 (0.41-0.86)^f^	0.48 (0.33-0.70)^e^
	Filipino	84.6 (4.4)^e^	51.4 (8.6)^e^	21.5 (9.3)^e^	0.29 (0.12-0.69)^f^	0.46 (0.21-1.00)^h^	0.18 (0.06-0.51)^f^
	East Asian	95.3 (1.8)	81.8 (6.1)	57.3 (10.8	1.07 (0.44-2.56)	2.32 (1.02-5.27)^g^	0.85 (0.32-2.27)
**Education**							
	<High school graduate	53.6 (9.3)^e^	29.0 (9.8)^e^	13.4 (5.5)^e^	0.04 (0.02-0.06)^e^	0.05 (0.29-0.10)^e^	0.08 (0.05-0.14)^e^
	High school graduate	81.4 (2.6)^e^	61.8 (2.6)^e^	36.0 (4.1)^e^	0.12 (0.08-0.18)^e^	0.16 (0.11-0.22)^e^	0.22 (0.16-0.32)^e^
	Some college/AA degree	92.3 (1.2)^e^	82.0 (1.2)^e^	60.9 (4.7)^e^	0.29 (0.20-0.43)^e^	0.39 (0.29-0.53)^e^	0.58 (0.42-0.81)^f^
	College graduate (ref)	98.4 (0.5)	92.1 (1.6)	74.2 (4.2)	(ref)	(ref)	(ref)
**Household income (US $)**							
	<$35,000 (ref)	73.0 (3.7)	62.8 (4.1)	35.7 (4.5)	1.00^i^	1.00^i^	1.00^i^
	$35,001-$50,000	84.4 (3.3)^e^	77.2 (4.4)^e^	58.1 (6.5)^e^	2.03 (1.44-2.97)^e^	1.75 (1.26-2.55)^e^	2.01 (1.40-2.91)^e^
	$50,001-$65,000	89.8 (2.9)^e^	85.3 (4.0)^e^	62.3 (7.8)^e^	3.31 (2.23-4.92)^e^	2.57 (1.72-3.86)^e^	1.92 (1.21-3.06)^f^
	$65,001-$80,000	94.6 (1.9)^e^	84.7 (3.9)^e^	63.6 (8.4)^e^	5.02 (3.25-7.77)^e^	2.19 (1.49-3.22)^e^	1.89 (1.21-2.95)^f^
	$80,001-$100,000	95.5 (1.6)^e^	90.4 (3.2)^e^	73.1 (±8.0)^e^	5.63 (3.60-8.80)^e^	2.91 (1.86-4.53)^e^	2.27 (1.39-3.70)^e^
	>$100,000	98.0 (0.6)^e^	92.5 (2.2)^e^	75.1 (7.1)^e^	8.75 (5.85-13.09)^e^	3.01 (1.97-4.61)^e^	2.29 (1.44-3.66)^e^
Model c-statistic^j^		—^k^	—	—	0.86	0.80	0.76

^a^MoE: 95% margin of error around estimated percentage (95% confidence intervals can be created from percentage ± MoE).

^b^AOR: adjusted odds ratio from logistic regression model that includes older age within age group, gender, race/ethnicity (including *Other Asian* and *Other* race/ethnicity categories), education, and household income.

^c^Younger age: 45-55 year, 66-70 year, 76-80 year; older age: 56-65 year, 71-75 year, 81-85 year.

^d^Ref: reference group for comparison of variable categories.

^e^Significantly differs from reference group at *P*<.001.

^f^Significantly differs from reference group at *P*<.01.

^g^Significantly differs from reference group at *P*<.05.

^h^Significantly differs from reference group at *P*<.051.

^i^95% CI not applicable.

^j^The model c-statistic assesses how well the full logistic regression model predicts which individuals use the internet to obtain information without help from other people.

^k^Not applicable.

**Table 4:** Use of Web-based health resources by middle-aged and older adults in the past year, all and internet users.

Web-based health resource		45 to 65 years, % (95% CI)	66 to 75 years, % (95% CI)	76 to 85 years, % (95% CI)
**Used health plan patient portal or Web-based HIA^a^** **resource**				
	All	69.0 (67.8-70.2)	68.1 (66.5-69.8)	52.2 (50.2-54.2)^b,c^
	Internet users^d^	72.1 (70.9-73.3)	77.4 (75.8-78.9)^b^	70.5 (68.3-72.6)^c^
**Used health plan’s patient portal to send email, check lab results, or order a prescription refill**				
	All	59.6 (58.3-60.9)	61.4 (59.7-63.1)	45.0 (43.0-47.0)^b,c^
	Internet users	62.3 (61.0-63.6)	69.7 (67.9-71.4)^b^	60.8 (58.5-63.0)^c^
**Obtained HIA from a Web-based source**				
	All	47.9 (46.6-49.2)	48.4 (46.6-50.2)	37.5 (35.6-39.5)^b,c^
	Internet users	50.1 (48.8-51.4)	55.2 (53.3-57.1)^e^	51.3 (48.9-53.6)

^a^HIA: health information or advice; includes having obtained health information in the past 12 months from any website or chat room/health community or having used a Web-based patient education program or podcast on the health plan’s website.

^b^Significantly (*P*<.001) higher or lower than the 45 to 65 years age group after controlling for gender and race/ethnicity.

^c^Significantly (*P*<.001) higher or lower than the 66 to 75 years age group after controlling for gender and race/ethnicity.

^d^Uses the internet by self or with someone else’s help.

^e^Significantly (*P*=.002) higher or lower than the 45 to 65 years age group after controlling for gender and race/ethnicity.

**Table 5:** Sociodemographic, health, and internet access characteristics differentiating self-reported users and nonusers of the health plan patient portal during the prior year, by age group.

Characteristic			45 to 65 years, weighted % (MoE^a^)		66 to 75 years, weighted % (MoE)		76 to 85 years, weighted % (MoE)		45 to 65 years, AOR^b^ (95% CI)		66 to 75 years, AOR (95% CI)		76 to 85 years, AOR (95% CI)
**Age group**													
	Younger age^c^ (ref^d^)	59.3 (2.1)		66.2 (2.5)		46.9 (2.7)		(ref)		(ref)		(ref)	
	Older age^c^	57.4 (1.9)^e^		59.3 (2.9)^f^		37.7 (5.1)^e^		1.19 (1.05-1.36)^e^		0.94 (0.78-1.15)		0.83 (0.61-1.13)	
**Gender**													
	Male (ref)	52.5 (2.2)		61.9 (2.7)		45.5 (3.5)		(ref)		(ref)		(ref)	
	Female	65.4 (1.9)^f^		64.8 (2.6)		40.9 (3.9)		1.83 (1.61-2.09)^f^		1.27 (1.04-1.54)^e^		1.17 (0.90-1.53)	
**Race/Ethnicity**													
	White non-Hispanic (ref)	64.7 (1.8)		67.9 (2.2)		48.1 (3.2)		(ref)		(ref)		(ref)	
	Black	49.9 (5.1)^f^		47.7 (8.0)^f^		26.2 (9.7)^f^		0.59 (0.47-0.75)^f^		0.59 (0.40-0.87)^e^		0.40 (0.22-0.75)^e^	
	Hispanic	52.1 (4.0)^f^		49.8 (7.0)^f^		19.5 (5.4)^f^		0.74 (0.60-.0.90)^e^		0.68 (0.48-0.96)^g^		0.41 (0.26-0.63)^f^	
	Filipino	47.0 (6.2)^f^		37.4 (8.3)^f^		23.7 (10.1)^f^		0.85 (0.53-1.37)		0.42 (0.24-1.14)		0.85 (0.25-2.91)	
	East Asian	54.4 (4.9)^f^		68.4 (7.4)		40.2 (10.9)		1.17 (0.76-1.82)		1.49 (0.69-3.21)		1.31 (0.42-4.06)	
**Education**													
	<High school graduate	30.4 (8.7)^f^		23.7 (8.8)^f^		19.5 (7.1)^f^		0.48 (0.30-0.76)^e^		0.48 (0.24-0.95)^g^		1.16 (0.62-2.20)	
	High school graduate	50.2 (3.4)^f^		48.2 (4.6)^f^		33.1 (5.2)^f^		0.70 (0.58-0.85)^f^		0.71 (0.53-0.96)^g^		1.11 (0.77-1.62)	
	Some college/AA degree	58.7 (2.5)^f^		64.7 (3.2)^g^		47.7 (4.8)^g^		0.77 (0.66-0.90)^f^		0.88 (0.70-1.11)		1.02 (0.74-1.41)	
	College graduate (ref)	64.9 (2.1)		72.3 (2.7)		55.3 (4.7)		(ref)		(ref)		(ref)	
**Household income (US $)**													
	≤$35,000 (ref)	44.1 (4.2)		48.7 (4.3)		29.6 (4.4)		(ref)		(ref)		(ref)	
	$35,001-$50,000	54.7 (4.6)^f^		61.0 (5.3)^f^		47.4 (6.5)^f^		1.31 (0.99-1.72)		1.16 (0.73-1.64)		1.33 (0.88-2.02)	
	$50,001-$65,000	58.2 (4.7)^f^		64.7 (5.7)^f^		52.8 (8.0)^f^		1.42 (1.07-1.88)^g^		1.15 (0.81-1.64)		1.40 (0.91-2.17)	
	$65,001-$80,000	56.7 (4.2)^f^		65.7 (5.4)^f^		51.1 (8.6)^f^		1.16 (0.89-1.51)		1.16 (0.82-1.64)		1.24 (0.73-2.09)	
	$80,001-$100,000	65.0 (3.7)^f^		67.7 (5.3)^f^		52.8 (9.4)^f^		1.67 (1.29-2.17)^f^		1.10 (0.77-1.57)		1.20 (0.72-2.01)	
	>$100,000	63.7 (2.3)^f^		75.5 (3.7)^f^		58.1 (8.0)^f^		1.54 (1.23-1.93)^f^		1.60 (1.47-2.30)^e^		1.39 (0.86-2.24)	
**Uses the internet to obtain information**													
	Does not use	2.2 (2.0)^f^		3.0 (2.2)^f^		2.9 (2.4)^f^		0.03 (0.01-0.06)^f^		0.02 (0.01-0.05)^f^		0.02 (0.01-0.04)^f^	
	Uses with someone’s help	33.4 (6.7)^f^		43.1 (6.3)^f^		41.6 (6.4)^f^		0.43 (0.31-0.60)^f^		0.35 (0.26-0.48)^f^		0.40 (0.29-0.56)^f^	
	Uses by self (ref)	62.8 (1.4)		73.4 (2.0)		66.4 (3.4)		(ref)		(ref)		(ref)	
**Has access to a computer**													
	No (ref)	13.3 (5.5)		8.0 (3.8)		7.0 (3.5)		(ref)		(ref)		(ref)	
	Yes	61.2 (1.5)^f^		68.6 (1.9)^f^		55.1 (3.0)^f^		2.33 (1.28-4.24)^e^		1.95 (0.97-3.95)		1.06 (0.52-2.14)	
**1 or more chronic condition**													
	No (ref)	49.3 (2.5)		58.1 (4.3)		35.7 (6.6)		(ref)		(ref)		(ref)	
	Yes	64.9 (1.7)^f^		64.9 (2.1)^e^		44.3 (2.9)^g^		2.22 (1.94-2.55)^f^		1.84 (1.47-2.30)^f^		1.76 (1.25-2.47)^e^	
Model c-statistic^h^			—^i^		—		—		0.71		0.77		0.82

^a^MoE: 95% margin of error around estimated percentage (95% confidence intervals can be created from percentage ± MoE).

^b^AOR: adjusted odds ratio from logistic regression model that includes older age within age group, gender, race/ethnicity (including other Asian and other race/ethnicity categories), education, household income, internet use, computer access, and 1 or more chronic condition.

^c^Younger age: 45 to 55 years, 66 to 70 years, 76 to 80 years; older age: 56 to 65 years, 71 to 75 years, 81 to 85 years.

^d^Ref: reference group for variable categories.

^e^Significantly differs from reference group at *P*<.01.

^f^Significantly differs from reference group at *P*<.001.

^g^Significantly differs from reference group at *P*<.05.

^h^The model c-statistic assesses how well the full logistic regression model predicts which individuals used the patient portal.

^i^Not applicable.

### Factors Associated with Patient Portal Use

Table 5 shows the relationship of sociodemographic, health, and internet access characteristics with use of the patient portal during the previous year. Within all age groups, there was no difference in patient portal use by self-rated health status (not shown) but having 1 or more chronic condition significantly increased portal use. Age, race/ethnicity, education, income, ability to use the internet without help, and having easy access to a computer were all significantly associated with patient portal use within all age groups. In the multivariable models, for all age groups, having 1 or more chronic condition and being able to use the internet without help significantly increased likelihood of having used the patient portal and having easy access to a computer increased likelihood of portal use among middle-aged adults and younger seniors. Educational attainment remained a significant factor for middle-aged adults and younger seniors, but not for older seniors. Within all age groups, blacks and Hispanics remained less likely than whites to have used the patient portal. A table showing AORs for age group­– specific models that include only the sociodemographic and chronic condition variables alongside AORs in Table 5 is found in Multimedia Appendix 2. Comparing across age groups, older seniors of all race/ethnicities were significantly (*P*<.05 or greater) less likely than middle-aged adults and younger seniors of the same race/ethnicity to have used the patient portal. Older seniors also had significantly lower rates of patient portal usage than the younger 2 groups at the high school graduate, some college, and college graduate levels; younger seniors with some college and college degrees had significantly higher usage than similarly educated middle-aged adults.

### Factors Associated with Use of Web-Based Health Information and Advice

Table 6 shows the associations of sociodemographic, health, and internet access factors with having used the internet to obtain HIA in the previous year. The overall percentages of adults who used Web-based HIA were lower than the percentages of those who used the patient portal, and disparities by race/ethnicity and levels of education and income were also not as large as found for patient portal use. In all age groups, Hispanics and Filipinos, adults with lower levels of education, and adults who needed help from another person to use the internet were less likely to have sought Web-based HIA. Among middle-aged adults, women were more likely to have sought HIA from a Web-based source than men, but the opposite was true among older seniors, with no gender difference observed among younger seniors. Similar to patient portal use, adults in all age groups who had 1 or more chronic condition were more likely to have sought Web-based HIA, but the rates of use did not differ by self-rated health status (not shown). In the multivariable models, lower educational attainment and requiring someone’s help to use the internet all significantly decreased likelihood of having sought Web-based HIA, whereas having 1 or more chronic condition increased likelihood. A table comparing AORs for models that include only the sociodemographic and chronic condition variables with AORs in Table 6 is available in Multimedia Appendix 3. Comparing across age groups, older seniors were significantly (*P*<.05 or greater) less likely than middle-aged adults and younger seniors of all race/ethnicities (with the exception of blacks for older vs younger seniors, where *P*<.08) to have sought Web-based HIA. Older seniors at lower levels of income were also significantly less likely than middle-aged and younger seniors.

### Age Group Differences in Internet, Patient Portal, and Web-Based Health Information and Advice Use Explained by Sociodemographic and Internet Access Factors

We examined whether the differences between middle-aged and senior age groups in internet use, patient portal use, and use of the internet to obtain health information shown in Tables 1 and 4 could be explained by differences in the characteristics of these groups using 3 sets of logistic regression models that compared the younger seniors to middle-aged adults, older seniors to younger seniors, and older seniors to middle-aged adults. Model 1 produced AORs adjusted for gender and race/ethnicity, and for patient portal and Web-based HIA use, having 1 or more chronic condition; Model 2 additionally adjusted for educational attainment and income; and Model 3 (for patient portal and Web-based HIA use only) added easy access to a computer and whether the person did not use the internet or required help from someone to use it. As is seen in Table 7, although differences in AORs between age groups for internet use substantially decrease after adjusting for education and income (Model 2) compared with adjusting for gender and race/ethnicity alone (Model 1), younger seniors remain significantly less likely than middle-aged adults, and older seniors significantly less likely than younger seniors, to be using the internet for purposes other than just email. The models for patient portal use show that additional adjustment for education, income, and 1 or more chronic health condition (Model 2) results in younger seniors being significantly more likely than middle-aged adults to have used the patient portal, whereas older seniors remain significantly less likely than younger seniors and middle-aged adults to have used the patient portal.

**Table 6:** Sociodemographic, health, and internet access characteristics differentiating self-reported users and nonusers of Web-based health information and advice resources during the prior year, by age group.

Characteristic		45 to 65 years, weighted % (MoE^a^)	66 to 75 years, weighted % (MoE)	76 to 85 years, weighted % (MoE)	45 to 65 years, AOR^b^ (95% CI)	66 to 75 years, AOR (95% CI)	76 to 85 years, AOR (95% CI)
**Age group**							
	Younger age^c^ (ref^d^)	47.0 (2.1)	51.5 (2.7)	38.8 (2.6)	(ref)	(ref)	(ref)
	Older age^c^	49.4 (1.9)	47.6 (2.9)	33.5 (5.0)	1.10 (0.97-1.25)	1.04 (0.87-1.24)	1.00 (0.75-1.35)
**Gender**							
	Male (ref)	43.3 (2.2)	49.9 (2.8)	41.7 (3.5)	(ref)	(ref)	(ref)
	Female	52.5 (2.0)^e^	49.9 (2.8)	32.3 (3.7)^e^	1.45 (1.28-1.64)^e^	1.06 (0.89-1.27)	0.85 (0.66-1.11)
**Race/Ethnicity**							
	White non-Hispanic (ref)	51.9 (1.9)	52.7 (2.4)	40.0 (3.2)	(ref)	(ref)	(ref)
	Black	48.6 (5.1)	41.7 (7.9)^f^	29.9 (10.0)	0.99 (0.78-1.25)	0.91 (0.61-1.35)	0.76 (0.42-1.38)
	Hispanic	39.6 (3.9)^e^	41.0 (6.9)^g^	19.3 (5.4)^e^	0.74 (0.71-0.89)^g^	0.85 (0.61-1.20)	0.60 (0.39-0.94)^f^
	Filipino	43.6 (6.1)^f^	32.8 (8.0)^e^	26.2 (10.6)^f^	0.70 (0.44-1.11)	0.71 (0.33-1.51)	1.95 (0.52-7.31)
	East Asian	38.2 (2.4)^f^	53.0 (8.1)	30.2 (10.0)	0.53 (0.34-0.82)^g^	1.30 (0.62-2.72)	1.70 (0.49-5.85)
**Education**							
	<High school graduate	21.1 (7.7)^e^	26.4 (9.3)^e^	20.2 (7.3)^e^	0.43 (0.26-0.72)^g^	0.74 (0.39-1.40)	1.46 (0.75-2.83)
	High school graduate	40.1 (3.3)^e^	32.6 (4.3)^e^	26.5 (5.0)^e^	0.76 (0.63-0.92)^g^	0.52 (0.40-0.68)^g^	0.92 (0.64-1.33)
	Some college/AA degree	49.1 (2.5)	51.5 (3.4)^e^	37.6 (4.6)^e^	0.91 (0.79-1.05)	0.88 (0.71-1.08)	0.81 (0.59-1.11)
	College graduate (ref)	52.2 (2.2)	58.0 (3.0)	50.1 (4.6)	(ref)	(ref)	(ref)
**Household income (US $)**							
	≤$35,000 (ref)	37.9 (4.1)	36.8 (4.2)	25.8 (2.2)	(ref)	(ref)	(ref)
	$35,001-$50,000	45.5 (4.6)^f^	48.5 (5.4)^e^	36.8 (3.2)^g^	1.15 (0.87-1.51)	1.24 (0.90-1.69)	1.04 (0.70-1.56)
	$50,001-$65,000	46.7 (4.7)^g^	51.6 (6.0)^e^	46.3 (4.2)^e^	1.15 (0.87-1.51)	1.34 (0.88-1.71)	1.34 (0.86-2.09)
	$65,001-$80,000	44.0 (4.2)^f^	54.9 (5.7)^e^	43.8 (4.3)^e^	0.91 (0.70-1.19)	1.38 (0.99-1.90)	1.24 (0.78-1.97)
	$80,001-$100,000	51.4 (3.8)^e^	54.6 (5.7)^e^	51.4 (4.8)^e^	1.23 (0.95-1.59)	1.19 (0.86-1.64)	1.55 (0.95-2.53)
	>$100,000	52.2 (2.4)^e^	57.1 (4.3)^e^	50.4 (4.1)^e^	1.28 (1.02-1.60)^f^	1.24 (0.92-1.66)	1.35 (0.84-2.17)
**Uses the internet to obtain information**							
	Does not use	0.0^h^	0.0^h^	0.2^h^	<0.01^h^	<0.01^h^	<0.01^h^
	Uses with someone’s help	28.0 (6.3)^e^	40.9 (6.3)^e^	40.2 (6.4)^e^	0.49 (0.35-0.68)^e^	0.59 (0.44-0.80)^e^	0.48 (0.34-0.67)^e^
	Uses by self (ref)	51.0 (1.5)	57.2 (2.2)	56.2 (3.5)	(ref)	(ref)	(ref)
**Has access to a computer**							
	No (ref)	10.6 (4.9)	9.7 (4.1)	6.8 (2.6)	(ref)	(ref)	(ref)
	Yes	49.6 (1.5)^e^	53.8 (2.1)^e^	46.5 (3.1)^e^	1.91 (0.99-3.68)	0.65 (0.31-1.36)	0.49 (0.25-0.97)^f^
**1 or more chronic condition**							
	No (ref)	39.2 (2.4)	45.4 (2.2)	27.7 (6.1)	(ref)	(ref)	(ref)
	Yes	53.1 (1.8)^e^	51.2 (1.1)^f^	38.1 (2.8)^g^	1.91 (1.67-2.18)^e^	1.47 (1.19-1.82)^e^	1.96 (1.38-2.78)^e^
Model c-statistics^i^		—^j^	—	—	0.66	0.7	0.79

^a^MoE: 95% margin of error around estimated percentage (95% confidence intervals can be created from percentage ± MoE).

^b^AOR: adjusted odds ratio from logistic regression model that includes older age within age group, gender, race/ethnicity (including other Asian and other race/ethnicity categories), education, household income, internet use, computer access, and 1 or more chronic condition.

^c^Younger age: 45 to 55 years, 66 to 70 years, 76 to 80 years; Older age: 56 to 65 years, 71 to 75 years, 81 to 85 years.

^d^Ref: reference group for comparison of variable categories.

^e^Significantly differs from reference group at *P*<.001.

^f^Significantly differs from reference group at *P*<.05.

^g^Significantly differs from reference group at *P*<.01.

^h^95% CI not applicable.

^i^The model c-statistic assesses how well the full logistic regression model predicts which individuals used a Web-based resource for health information or advice during the past year.

^j^Not applicable.

**Table 7:** Pairwise comparisons of middle-aged, younger senior, and older senior adult age groups’ use of the internet, patient portal, and Web-based health information resources after adjusting for sociodemographic and other characteristics (Model 1 includes gender, race/ethnicity, and 1 or more chronic condition for patient portal and Web-based health information and advice use only; Model 2 includes gender, race/ethnicity, education, income, and 1 or more chronic condition for patient portal and Web-based health information and advice use only; and Model 3, for patient portal and Web-based health information use only, includes gender, race/ethnicity, education, income, 1 or more chronic condition, whether uses internet with help or does not use the internet, and whether has easy access to a computer. The age group listed after the “vs” is the referent group for the age group comparisons).

Internet resource and age group comparisons		Model 1, AOR^a^ (95% CI)	Model 2, AOR (95% CI)	Model 3, AOR (95% CI)
**Internet user (with or without help)^b^**				
	66-75 years vs 45-65 years	0.31 (0.25-0.37)^c^	0.40 (0.33-0.50)^c^	—^d^
	76-85 years vs 66-75 years	0.25 (0.21-0.30)^c^	0.34 (0.28-0.42)^c^	—
	76-85 years vs 45-65 years	0.08 (0.06-0.10)^c^	0.15 (0.12-0.18)^c^	—
**Used patient portal in the past 12 months**				
	66-75 years vs 45-65 years	1.02 (0.91-1.13)	1.14 (1.02-1.27)	1.42 (1.26-1.61)^c^
	76-85 years vs 66-75 years	0.41 (0.35-0.47)^c^	0.50 (0.43-0.58)^c^	0.75 (0.63-0.89)^c^
	76-85 years vs 45-65 years	0.40 (0.35-0.45)^c^	0.54 (0.46-0.62)^c^	1.01 (0.86-1.20)
**Obtained health information from a Web-based source in the past 12 months**				
	66-75 years vs 45-65 years	0.95 (0.85-1.05)	1.02 (0.92-1.10)	1.18 (1.05-1.32)^e^
	76-85 years vs 66-75 years	0.56 (0.49-0.64)^c^	0.68 (0.59-0.78)^c^	0.95 (0.81-1.12)
	76-85 years vs 45-65 years	0.51 (0.45-0.59)^c^	0.66 (0.58-0.76)^c^	1.15 (0.98-1.35)

^a^AOR: adjusted odds ratio; 95% CI: 95% confidence interval around AOR.

^b^AORs for internet use without help were virtually the same as those shown for internet use with or without help.

^c^Significantly different from referent age group at *P*<.001.

^d^Not applicable.

^e^Significantly different from referent age group at *P*<.05.

Additional adjustment for internet user status and computer access (Model 3) increases likelihood of patient portal use in the prior year by younger seniors compared with middle-aged adults, significantly reduces differences between older and younger seniors, and removes differences between older seniors and middle-aged adults. Similar results are seen for use of Web-based health information resources.

## Discussion

### Principal Findings

To our knowledge, ours is the first study to examine prevalence and factors influencing use of Web-based resources for health information and patient portals separately for middle-aged adults, younger seniors, and older seniors. Previous studies have shown that age, race/ethnicity, educational attainment, and low income are social determinants of being an internet user [11] and patient portal user [26-28] in adult and older adult populations. Our study showed that these factors operate similarly within middle-aged, younger senior, and older senior age groups to create disparities in use of the internet, use of the internet without help, and use of patient portals and Web-based health information resources. Furthermore, we showed that these same social determinants were associated with disparities in use of the internet without help from another person and use of Web-based health resources even among adults who used the internet. Our study also showed that differences between middle-aged and older age groups in prevalence of use of Web-based health resources were not fully explained by group level differences in sociodemographic, health, and internet access characteristics. By including information about the percentages of adults in different race/ethnic groups and at different levels of education and income who used the internet and 2 Web-based health resources alongside the results of the multivariable logistic regression models showing the independent relationship of these factors to use of these resources, we provide context for interpretation of the bivariate and multivariable associations of these factors with use of Web-based resources that is not generally been found in previous publications on this topic.

In our study, we found significant differences in prevalence of internet use and use of Web-based health resources across and within our middle-aged and older adult age groups. We found that the percentage of internet users aged between 45 and 55 years approximated that of younger adults and, similar to other studies [29], that the percentage of baby boomers (aged between 66 and 70 years) who use the internet did not significantly differ from that of older middle-aged adults (aged between 56 and 65 years). However, above the age of 70 years, we found steep declines in internet use, such that although over 90% of the youngest seniors were using the internet, less than two-thirds of those aged 81 to 85 years were doing so. We also showed that in contrast to younger adults, starting at middle age, there is an increasingly wide gap between the percentages of adults who use internet resources and the percentages who are able to do so without help from another person. This gap in how the internet is accessed has implications for how national and population-based surveys should consider measuring internet access, as well as for assessing progress toward meeting Healthy People 2020 goals for use of health information technologies.

Across all age groups, adults who used the internet but needed help from another person to do so were significantly less likely to report using the patient portal and Web-based health information resources than those who used the internet without someone’s help. This disparity persisted even after controlling for education and presence of a chronic health condition, a factor that significantly increased likelihood of individuals using the patient portal and seeking HIA from a Web-based source in our multivariable analyses. The gap in skills and comfort in using the internet, which has been termed e-literacy or *digital readiness*, can potentially be remedied by motivational interviewing and demonstration of the advantages of using Web-based health resources to increase patient engagement, offering training (in-person, Web-based, and print materials) in how to use patient portal features and search for health information using the internet, and providing ongoing encouragement for use of Web-based resources.

In addition to e-literacy issues, access to high-speed internet and digital tools that make it possible for middle-aged and older adults to comfortably navigate the internet can also be a barrier to the use of Web-based health resources. In our study population, we found that one-third of middle-aged and older adults who do not use the internet at all and 42% of adults who need help from another person to use the internet do not have easy access to a computer. Although a growing percentage of adults own internet-enabled smartphones, in the short term, smartphones are unlikely to be used to interact with patient portals and to search for Web-based health information by middle-aged and older adults who lack easy access to an internet-enabled computer or tablet. A national survey of US adults found that in 2018, the use of internet-enabled smartphones remained significantly lower among middle-aged adults (73%) and seniors (49%) than younger adults (>90%), with less than one-third of adults ≥75 years and owning a smartphone [30]. Middle-aged and older adult smartphone owners primarily use their devices for phone calls, text messaging, and emailing [31]. In addition to the lack of experience and confidence, many older adults find it difficult to read information on small smartphone screens, use small virtual keyboards and touch screens to navigate the internet, and interact with apps [31]. Thus, increasing engagement with Web-based health resources may require helping those who do not have a computer or high-speed internet at home identify affordable laptop computers that they can take to venues offering free Wi-Fi, low-cost home internet plans, or other locations such as public libraries or friends’ homes where they can use these tools with sufficient privacy. In situations requiring home internet, such as telemonitoring, it may be necessary to provide a computer and cover internet fees as a durable medical equipment benefit.

Consistent with previous research [29,32-34], we found that middle-aged and older adults with lower incomes and lower educational attainment were significantly less likely to be using the internet and using the internet for health-related purposes than those with higher incomes and higher educational attainment. Both low educational attainment and low income remained significant independent predictors of lower use of the internet and Web-based health resources in our multivariable models. Middle-aged and older adults who did not attend college are more likely to be *digitally unprepared* [35] than those with higher education as they are less likely to have had the opportunity to learn how to use computers and the internet while in school or at work. In addition, lower income adults, and especially older adults on fixed incomes, may not be able to afford high-speed home internet and a computer with sufficient memory and speed to interact with graphic-rich websites and streamed video content [31] or consider these digital technologies to be priority expenses. In our health plan survey, we found that adults with incomes of ≤$35,000 were not only less likely to be internet users than those at higher income levels, but also that the percentages who had easy access to a computer in this income group (86% of middle-aged adults, 78% of younger seniors, and 57% of older seniors) were significantly lower than those of similarly aged adults in the next higher income level. Similarly, computer access was significantly lower among those who had not graduated from high school (76% of middle-aged adults, 56% of younger seniors, and 42% of older seniors) than among those at higher levels of education. These findings suggest that the transition of health plans and government agencies to the use of secure portals and websites for dissemination of health information could potentially limit, rather than improve, the ability of less educated and lower income middle-aged and older adults to get health information and patient education and communicate with their health care providers in a way that feels comfortable.

Finally, we found that although having 1 or more chronic health condition was not significantly associated with being an internet user, this factor did significantly increase likelihood of middle-aged and older adults using the patient portal and obtaining health information from a Web-based source. This finding is unsurprising, given that adults with chronic health conditions would have more reason to use the patient portal to communicate with their health care providers, look up test results, and order prescription refills, and to obtain information and advice to manage their health conditions and health care. However, it does suggest that when possible, future studies should examine patient portal and Web-based health information use for the subpopulation of adults with a chronic health condition in addition to use in the broader population, especially when comparing use of these Web-based health resources across different demographic groups or over time.

### Strengths

This study has several strengths. We used a large sociodemographically diverse dataset to examine the association of several social determinants with being an internet user and using 2 different types of internet-based health resources, a health plan patient portal and Web-based health information, separately for middle-aged, younger senior, and older senior adults. We showed how prevalence of internet use and use of Web-based health resources varied by race/ethnicity, educational attainment, income, and health status within and across these age groups in addition to evaluating the independent effects of these factors within age groups using multivariable models. Our ability to differentiate adults who were able to use the internet on their own from those who required help from another person and adults who had and did not have easy access to a computer enabled us to examine how these factors varied across age groups and affected use of Web-based health resources independent of sociodemographic factors.

### Limitations

The survey was conducted with adults from 1 Northern California health plan membership that, although fairly representative of Northern California adults, is not representative of the US adult population with regard to educational attainment, income, broadband internet access, and access to a comprehensive health plan website. For over a decade, Kaiser Permanente has extensively promoted the use of its kp.org website to members as a resource to obtain HIA and the use of its patient portal to communicate with clinicians, view lab test results, order prescriptions refills, and access other health care–related information in the member’s electronic health record. In addition, the health plan membership surveyed for this study was better educated (higher percentages of college graduates in each age group) and better off financially (lower percentages with household income <$35,000) than the overall US adult population around the time of the survey [36], had English as a primary language, and resided primarily in communities with good broadband coverage. The confluence of these factors would be expected to inflate the percentages of adults in all 3 age groups who used DITs and obtained health information from a Web-based source. In fact, compared with estimates for ages 45 to 65, 66 to 75, and 76 to 85 years in the US population from the 2015 Current Population Survey Computer and Internet Use Supplement, health plan adults in the same age groups were significantly more likely to be using the internet (approximately 96% vs 76%, 89% vs 64%, and 69% vs 42%, respectively), using the internet to obtain health information (48% vs 40%, 48% vs 31%, and 37% vs 20%, respectively), and using the internet to communicate or obtain health information from a health care provider (60% vs 22%, 61% vs 18%, and 45% vs 10%, respectively) [36]. Our patient portal use and Web-based health information resource use variables were based on self-report, which may have led to over- or under-reporting of use of these resources. However, most population surveys and studies of internet use for health purposes have similarly relied on self-reported data to study this topic. Survey response bias may also have resulted in overestimation of Web-based health resource use if adults who were better-educated, nonminority, and had 1 or more chronic condition were more likely to participate in the survey. Unfortunately, the post-stratification survey weighting factor did not adjust for these factors. Finally, although we were able to examine for race/ethnic disparities in use of the internet, patient portal, and Web-based health information resources, the survey dataset limited our ability to examine how gender, educational attainment, income, and internet use factors may differentially influence use of Web-based health resources by middle-aged and older adults of different racial/ethnic groups. Future research is needed on how these and other sociocultural factors influence uptake of DITs for health purposes within specific race/ethnic groups.

### Conclusions

The internet offers a low-cost and effective method to access personal health information from secure patient portals and general health information from nonsecure websites. Web-based health information seeking behavior has been shown to help patients with chronic conditions become more knowledgeable and engaged with their health care, as well as better connected with resources to help them manage and cope with health-related concerns. Yet for many middle-aged and older adults, lack of education, financial strain, lack of computer and high-speed internet access, and inadequate skills and experience with regard to DITs will be barriers to their benefiting from eHealth information resources. As health plans begin to rely more heavily on patient portals and websites to communicate health-related information, digital divides between adults with lower levels of education and income and those with higher levels of education and income may negatively impact easy access to health information and patient education and make health care communications more difficult for middle-aged and older adults in vulnerable sociodemographic groups. This in turn may exacerbate disparities in health and health care use.

Health plans and other health organizations that want to serve the health information needs of all segments of a diverse population should take into consideration that middle-aged and older adults, especially those who are less educated and less affluent, are less likely than younger adults to engage with Web-based resources unless these resources are perceived to have demonstrable benefits over more traditional methods, to be convenient and easy to use, and to add to, not diminish, opportunities for social interaction with their health care team when this is a valued part of health care and the patient-provider relationship. Increasing use of patient portals and Web-based health information resources by these adults will require government agencies and health plans to make sure that their websites are easy to navigate and the content easy to view by aging adults, many of whom will have less visual acuity, motor coordination, digital experience, and access to sophisticated digital technology than the younger adults who often are responsible for developing these websites. Furthermore, middle-aged and older adults, especially those who are not already digitally engaged, will need active encouragement and support from health care providers to use Web-based health resources. Finally, health plans and government agencies offering patient portals and websites must continue to offer traditional patient counseling and information services to accommodate all patient preferences and needs.

## Appendix

Multimedia Appendix 1 Sociodemographic factors differentiating middle-aged and older internet-using adults who use the internet by themselves to get information from websites.

Multimedia Appendix 2 Comparison of logistic regression models of factors predicting self-reported use of the health plan patient portal during the 12 months prior to the survey, by age group.

Multimedia Appendix 3 Comparison of logistic regression models of factors predicting self-report of having obtained health information or advice from a Web-based resource during the 12 months prior to the survey, by age group.

## Abbreviations

AOR: adjusted odds ratio
COPD: chronic obstructive pulmonary disease
DIT: digital information technology
eHealth: electronic health
HIA: health information and advice
KPNC: Kaiser Permanente in Northern California
MHS: Member Health Survey
MoE: margin of error
