# Supplementary material for: Prevalence and Factors Influencing Use of Internet and Electronic Health Resources by Middle-Aged and Older Adults in a US Health Plan Population: Cross-Sectional Survey Study
Source: JMIR Aging. 2019 Mar 26;2(1):e11451. doi: 10.2196/11451 (PMC6715345; doi:10.2196/11451)
Supplement: Multimedia Appendix 1 [file aging_v2i1e11451_app1.pdf]

Multimedia Appendix 1. Sociodemographic factors differentiating middle-aged and older internet-using adults who use the internet by themselves to get information from websites

	45-65 yr	66-75 yr	76-85 yr	45-65 yr	66-75 yr	76-85 yr
	**Wtd.% (MoE)**	**Wtd.% (MoE)**	**Wtd.% (MoE)**	**AOR (95% CI)**	**AOR (95% CI)**	**AOR (95% CI)**
**Age group**						
Younger age[a] (ref)	96.5% (±0.8)	90.8% (±1.5)	78.8% (±2.5)	(ref)	(ref)	(ref)
Older age[a]	94.8% (±0.9) [b]	88.9% (±1.9)	73.7% (±5.6)	0.55 (0.40-0.76) [c]	0.93 (0.69-1.25)	0.76 (0.54-1.08)
**Gender**						
Male (ref)	94.9% (±1.0)	90.6% (±1.7)	77.5% (±3.5)	(ref)	(ref)	(ref)
Female	96.5% (±0.7) [b]	89.6% (±1.7)	76.2%(±4.0)	1.85 (1.34-2.54) [c]	1.11 (0.82-1.51)	1.18 (0.84-1.66)
**Race/Ethnicity**						
White non-Hispanic (ref)	97.0% (±0.7)	92.4% (±1.3)	80.2% (±2.9)	(ref)	(ref)	(ref)
Black	93.7% (±2.7) [b]	89.0% (±5.6)	61.7% (±13.3) [b]	0.56 (0.32-0.96) [d]	0.92 (0.50-1.69)	0.46 (0.26-0.82) [b]
Hispanic	92.2% (±2.2) [c]	85.4% (±5.4) [b]	62.9% (±9.6) [c]	0.61 (0.40-0.92) [d]	0.71 (0.42-1.18)	0.61 (0.37-1.01)
Filipino	90.2% (±3.9) [d]	67.6% (±9.1) [d]	34.8% (±14.6) [b]	0.29 (0.10-0.86) [d]	0.75 (0.33-1.70)	0.07 (0.02-0.30) [c]
East Asian	97.3% (±1.4)	91.6% (±4.8)	83.4% (±9.1)	1.17 (0.39-3.54)	4.53 (1.74-11.77) [b]	0.68 (0.16-2.97)
**Education**						
<High school graduate	66.4% (±10.4) [c]	50.5% (±14.1) [c]	35.9% (±12.9) [c]	0.03 (0.01-0.05) [c]	0.06 (0.03-0.13) [c]	0.13 (0.07-0.25) [c]
High school graduate	90.1% (±2.2) [c]	79.9% (±4.1) [c]	68.2% (±6.5) [c]	0.10 (0.06-0.17) [c]	0.20 (0.13-0.32) [c]	0.40 (0.25-0.63) [c]
Some college/AA degree	95.7% (±1.0) [c]	90.3% (±2.1) [c]	80.8% (±4.0) [c]	0.24 (0.15-0.39) [c]	0.43 (0.28-0.66) [c]	0.79 (0.51-1.21)
College graduate (ref)	99.2% (±0.3)	95.2% (±1.3)	83.3% (±3.9)	(ref)	(ref)	(ref)
**Household income (US $)**						
≤ $35,000 (ref)	86.9% (±3.2)	82.5% (±3.7)	67.8% (±5.8)	(ref)	(ref)	(ref)
$35,001 - $50,000	92.5% (±2.5) [c]	87.8% (±3.7) [c]	79.5% (±6.0) [b]	2.01 (1.24-3.27) [b]	1.18 (0.74-1.88)	1.46 (0.89-2.39)
$50,001 - $65,000	92.6% (±2.6) [c]	92.0% (±3.2) [c]	75.5% (±7.9)	2.00 (1.21-3.31) [b]	1.74 (1.02-2.97) [d]	1.05 (0.61-1.91)
$65,001 - $80,000	96.9% (±1.5) [c]	89.4% (±3.3) [c]	79.1% (±8.2) [d]	3.81 (2.11-6.89) [c]	1.13 (0.71-1.81)	1.13 (0.62-2.04)
$80,001 - $100,000	96.8% (±1.5) [c]	94.6% (±2.6) [c]	85.3% (±6.6) [c]	3.35 (1.87-5.99) [c]	1.93 (1.04-3.61) [d]	1.65 (0.88-3.07)
> $100,000	98.7% (±0.5) [c]	95.6% (±1.7) [c]	83.4% (±6.5) [b]	5.41 (3.21-9.12) [c]	2.05 (1.17-3.62) [d]	1.24 (0.69-2.20)
**Model c-statistic [e]**	--	--	--	c=.83	c=.76	c=.70

Wtd. % = weighted percentage; MoE = 95% margin of error around estimated percentage (95% confidence intervals can be created from percentage ± MoE); AOR=adjusted odds ratio from logistic regression model that includes age group, gender, race/ethnicity (including other Asian and other race/ethnicity categories), education, and household income; ref = reference group for comparison of variable categories; --: not applicable.

[a] Younger age: 45-55 yr, 66-70 yr, 76-80 yr; Older age: 56-65 yr, 71-75 yr, 81-85 yr

[b] Significantly differs from reference group at p<.01

[c] Significantly differs from reference group at p<.001

[d] Significantly differs from reference group at p<.05

[e] The model c-statistic assesses how well the full logistic regression model predicts who uses the internet by themself.
