# Supplementary material for: Prevalence and Factors Influencing Use of Internet and Electronic Health Resources by Middle-Aged and Older Adults in a US Health Plan Population: Cross-Sectional Survey Study
Source: JMIR Aging. 2019 Mar 26;2(1):e11451. doi: 10.2196/11451 (PMC6715345; doi:10.2196/11451)
Supplement: Multimedia Appendix 2 [file aging_v2i1e11451_app2.pdf]

Multimedia Appendix 2. Comparison of logistic regression models of factors predicting self-reported use of the health plan patient portal during the 12 months prior to the survey, by age group

	**45-65 yr**		**66-75 yr**		**76-85 yr**	
	**Model 1**	**Model 2**	**Model 1**	**Model 2**	**Model 1**	**Model 2**
	**AOR (95% CI)**	**AOR (95% CI)**	**AOR (95% CI)**	**AOR (95% CI)**	**AOR (95% CI)**	**AOR (95% CI)**
**Age group**						
Younger age [a] (ref)	(ref)	(ref)	(ref)	(ref)	(ref)	(ref)
Older age [a]	1.08 (0.96-1.23)	1.19 (1.05-1.36) [b]	0.82 (0.68-0.97) [d]	0.94 (0.78-1.15)	0.69 (0.53-0.90) [b]	0.83 (0.61-1.13)
**Gender**						
Male (ref)	(ref)	(ref)	(ref)	(ref)	(ref)	(ref)
Female	1.88 (1.66-2.14) [b]	1.83 (1.61-2.09) [c]	1.29 (1.08-1.55) [b]	1.27 (1.04-1.54) [b]	1.06 (0.84-1.34)	1.17 (0.90-1.53)
**Race/Ethnicity**						
White non-Hispanic (ref)	(ref)	(ref)	(ref)	(ref)	(ref)	(ref)
Black	0.55 (0.44-0.68) [c]	0.59 (0.47-0.75) [c]	0.50 (0.35-0.71) [c]	0.59 (0.40-0.87) [b]	0.40 (0.22-0.72) [b]	0.40 (0.22-0.75) [b]
Hispanic	0.69 (0.57-0.84) [c]	0.74 (0.60-.0.90) [b]	0.61 (0.44-0.83) [b]	0.68 (0.48-0.96) [d]	0.34 (0.23-0.50) [c]	0.41 (0.26-0.63) [c]
Filipino	0.77 (0.44-1.22)	0.85 (0.53-1.37)	0.39 (0.19-0.79) [b]	0.42 (0.24-1.14)	0.61 (0.21-1.79)	0.85 (0.25-2.91)
East Asian	1.17 (0.76-1.82)	1.17 (0.76-1.82)	1.47 (0.73-2.95)	1.49 (0.69-3.21)	1.15 (0.42-3.14)	1.31 (0.42-4.06)
**Education**						
<High school graduate	0.28 (0.18-0.43) [c]	0.48 (0.30-0.76) [b]	0.17 (0.10-0.30) [c]	0.48 (0.24-0.95) [d]	0.31 (0.18-0.53) [c]	1.16 (0.62-2.20)
High school graduate	0.57 (0.48-0.69) [c]	0.70 (0.58-0.85) [c]	0.43 (0.33-0.55) [c]	0.71 (0.53-0.96) [d]	0.48 (0.34-0.66) [c]	1.11 (0.77-1.62)
Some college/AA degree	0.74 (0.64-0.86) [c]	0.77 (0.66-0.90) [c]	0.73 (0.59-0.91) [b]	0.88 (0.70-1.11)	0.78 (0.59-1.05) [b]	1.02 (0.74-1.41)
College graduate (ref)	(ref)	(ref)	(ref)	(ref)	(ref)	(ref)
**Household income (US $)**						
≤ $35,000 (ref)	(ref)	(ref)	(ref)	(ref)	(ref)	(ref)
$35,001 - $50,000	1.57 (1.21-2.03) [c]	1.31 (0.99-1.72)	1.51 (1.12-2.04) [b]	1.16 (0.73-1.64)	1.87 (1.32-2.66) [c]	1.33 (0.88-2.02)
$50,001 - $65,000	1.91 (1.47-2.49) [c]	1.42 (1.07-1.88) [d]	1.64 (1.19-2.25) [b]	1.15 (0.81-1.64)	2.09 (1.37-3.18) [c]	1.40 (0.91-2.17)
$65,001 - $80,000	1.61 (1.25-2.07) [c]	1.16 (0.89-1.51)	1.66 (1.21-2.27) [b]	1.16 (0.82-1.64)	1.78 (1.17-2.72) [d]	1.24 (0.73-2.09)
$80,001 - $100,000	2.35 (1.84-3.00) [c]	1.67 (1.29-2.17) [c]	1.59 (1.16-2.19) [b]	1.10 (0.77-1.57) [b]	1.75 (1.10-2.79) [b]	1.20 (0.72-2.01)
> $100,000	2.16 (1.74-2.66) [c]	1.54 (1.23-1.93) [c]	2.20 (1.63-2.98) [c]	1.60 (1.47-2.30)	2.10 (1.35-3.27) [b]	1.39 (0.86-2.24)
**Uses the internet to get information**						
Does not use		0.03 (0.01-0.06) [c]		0.02 (0.01-0.05) [c]		0.02 (0.01-0.04) [c]
Use's with someone's help		0.43 (0.31-0.60) [c]		0.35 (0.26-0.48) [c]		0.40 (0.29-0.56) [c]
Uses by self (ref)		(ref)		(ref)		(ref)
**Has access to a computer**						
No (ref)		(ref)		(ref)		(ref)
Yes		2.33 (1.28-4.24) [b]		1.95 (0.97-3.95)		1.06 (0.52-2.14)
**1 or more chronic conditions**						
No (ref)	(ref)	(ref)	(ref)	(ref)	(ref)	(ref)
Yes	2.14 (1.87-2.45) [c]	2.22 (1.94-2.55) [c]	1.57 (1.27-1.94) [c]	1.84 (1.47-2.30) [c]	1.69 (1.22-2.33) [b]	1.76 (1.25-2.47) [b]
**Model c-statistic**	c=.67	c=0.71	c=.70	c=0.77	c=.70	c=0.82

Model 1 includes sociodemographic factors only; Model 2 adds health and internet access factors; AOR= adjusted odds ratio; CI= 95% confidence interval around AOR; ref = reference group for comparison of variable categories.

[a] Younger age: 45-55 yr, 66-70 yr, 76-80 yr; Older age: 56-65 yr, 71-75 yr, 81-85 yr

[b] Significantly differs from reference group at $p<.01$

[c] Significantly differs from reference group at $p<.001$

[d] Significantly differs from reference group at $p<.05$

[e] The model c-statistic assesses how well the full logistic regression model predicts who reported using the patient portal during the prior year.
