# Supplementary material for: Prevalence and Factors Influencing Use of Internet and Electronic Health Resources by Middle-Aged and Older Adults in a US Health Plan Population: Cross-Sectional Survey Study
Source: JMIR Aging. 2019 Mar 26;2(1):e11451. doi: 10.2196/11451 (PMC6715345; doi:10.2196/11451)
Supplement: Multimedia Appendix 3 [file aging_v2i1e11451_app3.pdf]

Multimedia Appendix 3. Comparison of logistic regression models of factors predicting self-report of having obtained health information or advice from a Web-based resource during the 12 months prior to the survey, by age group

	**45-65 yr**		**66-75 yr**		**66-75 yr**	**76-85 yr**
	**Model 1**	**Model 2**	**Model 1**	**Model 2**	**Model 1**	**Model 2**
	**AOR (95% CI)**	**AOR (95% CI)**	**AOR (95% CI)**	**AOR (95% CI)**	**AOR (95% CI)**	**AOR (95% CI)**
**Age group**						
Younger age[a] (ref)	(ref)	(ref)	(ref)	(ref)	(ref)	(ref)
Older age [a]	1.09 (0.97-1.23)	1.10 (0.97-1.25)	0.93 (0.79-1.10)	1.04 (0.87-1.24)	0.83 (0.64-1.08)	1.00 (0.75-1.35)
**Gender**						
Male (ref)	(ref)	(ref)	(ref)	(ref)	(ref)	(ref)
Female	1.52 (1.35-1.71) [b]	1.45 (1.28-1.64) [b]	1.10 (0.93-1.30)	1.06 (0.89-1.27)	0.84 (0.66-1.06)	0.85 (0.66-1.11)
**Race/Ethnicity**						
White non-Hispanic (ref)	(ref)	(ref)	(ref)	(ref)	(ref)	(ref)
Black	0.94 (0.75-1.17)	0.99 (0.78-1.25)	0.76 (0.53-1.10)	0.91 (0.61-1.35)	0.70 (0.41-1.21)	0.76 (0.42-1.38)
Hispanic	0.70 (0.58-0.85) [b]	0.74 (0.71-0.89) [c]	0.77 (0.56-1.05)	0.85 (0.61-1.20)	0.47 (0.32-0.69) [b]	0.60 (0.39-0.94) [d]
Filipino	0.71 (0.45-1.12)	0.70 (0.44-1.11)	0.52 (0.25-1.08)	0.71 (0.33-1.51)	1.42 (0.44-4.59)	1.95 (0.52-7.31)
East Asian	0.55 (0.36-0.85) [c]	0.53 (0.34-0.82) [c]	1.20 (0.59-2.44)	1.30 (0.62-2.72)	1.40 (0.44-4.29)	1.70 (0.49-5.85)
**Education**						
<High school graduate	0.30 (0.19-0.49) [b]	0.43 (0.26-0.72) [c]	0.33 (0.20-0.56) [b]	0.74 (0.39-1.40)	0.39 (0.23-0.66) [b]	1.46 (0.75-2.83)
High school graduate	0.67 (0.56-0.80) [b]	0.76 (0.63-0.92) [c]	0.38 (0.30-0.49) [b]	0.52 (0.40-0.68) [b]	0.44 (0.32-0.62) [b]	0.92 (0.64-1.33)
Some college/AA degree	0.87 (0.75-1.00)	0.91 (0.79-1.05)	0.78 (0.64-0.95) [d]	0.88 (0.71-1.08)	0.67 (0.51-0.89) [c]	0.81 (0.59-1.11)
College graduate (ref)	(ref)	(ref)	(ref)	(ref)	(ref)	(ref)
**Household income (US $)**						
≤ $35,000 (ref)	(ref)	(ref)	(ref)	(ref)	(ref)	(ref)
$35,001 - $50,000	1.32 (1.02-1.70) [d]	1.15 (0.87-1.51)	1.49 (1.12-1.99) [c]	1.24 (0.90-1.69)	1.45 (1.02-2.07) [d]	1.04 (0.70-1.56)
$50,001 - $65,000	1.43 (1.10-1.85) [c]	1.15 (0.87-1.51)	1.57 (1.15-2.14) [c]	1.34 (0.88-1.71)	1.91 (1.23-2.96) [c]	1.34 (0.86-2.09)
$65,001 - $80,000	1.17 (0.91-1.51)	0.91 (0.70-1.19)	1.76 (1.30-2.39) [b]	1.38 (0.99-1.90)	1.62 (1.08-2.42) [d]	1.24 (0.78-1.97)
$80,001 - $100,000	1.58 (1.24-2.00) [b]	1.23 (0.95-1.59)	1.54 (1.13-2.10) [c]	1.19 (0.86-1.64)	2.00 (1.26-3.15) [c]	1.55 (0.95-2.53)
> $100,000	1.58 (1.28-1.96) [b]	1.28 (1.02-1.60) [d]	1.58 (1.20-2.08) [c]	1.24 (0.92-1.66)	1.91 (1.21-3.01) [c]	1.35 (0.84-2.17)
**Uses the internet to get information**						
Does not use		<0.01 (--) [b]		<0.01 (--) [b]		<0.01 (--) [b]
Uses with someone's help		0.49 (0.35-0.68) [b]		0.59 (0.44-0.80) [b]		0.48 (0.34-0.67) [b]
Uses by self (ref)		(ref)		(ref)		(ref)
**Has access to a computer**						
No (ref)		(ref)		(ref)		(ref)
Yes		1.91 (0.99-3.68)		0.65 (0.31-1.36)		0.49 (0.25-0.97) [d]
**1 or more chronic conditions**						
No (ref)	(ref)	(ref)	(ref)	(ref)	(ref)	(ref)
Yes	1.86 (1.64-2.12) [b]	1.91 (1.67-2.18) [b]	1.38 (1.13-1.69) [c]	1.47 (1.19-1.82) [b]	1.85 (1.32-2.59) [b]	1.96 (1.38-2.78) [b]
**Model c-statistic**	c=0.63	c=0.66	c=0.64	c=0.70	c=0.68	c=0.79

Model 1 includes sociodemographic factors only; Model 2 adds health and internet access factors; AOR= adjusted odds ratio; CI= 95% confidence interval around AOR; ref = reference group for comparison of variable categories.

[a] Younger age: 45-55 yr, 66-70 yr, 76-80 yr; Older age: 56-65 yr, 71-75 yr, 81-85 yr

[b] Significantly differs from reference group at $p<.001$

[c] Significantly differs from reference group at $p<.01$

[d] Significantly differs from reference group at $p<.05$

[e] The model c-statistic assesses how well the full logistic regression model predicts who reported obtaining Web-based health information or advice during the prior year.
